# Nasopharyngeal Carcinoma Signaling Pathway: An Update on Molecular Biomarkers

**DOI:** 10.1155/2012/594681

**Published:** 2012-03-05

**Authors:** Warut Tulalamba, Tavan Janvilisri

**Affiliations:** ^1^Graduate Program in Molecular Medicine, Faculty of Science, Mahidol University, Rama VI Road, Bangkok 10400, Thailand; ^2^Department of Biology, Faculty of Science, Mahidol University, Rama VI Road, Bangkok 10400, Thailand

## Abstract

Nasopharyngeal carcinoma (NPC) is an uncommon cancer, which has a distinctive ethnic and geographic distribution. Etiology of NPC is considered to be related with a complex interaction of environmental and genetic factors as well as Epstein-Barr virus infection. Since NPC is located in the silent painless area, the disease is usually therefore diagnosed at the advanced stages; hence early detection of NPC is difficult. Furthermore, understanding in molecular pathogenesis is still lacking, pondering the identification of effective prognostic and diagnostic biomarkers. Dysregulation of signaling molecules in intracellular signal transduction, which regulate cell proliferation, apoptosis, and adhesion, underlines the basis of NPC pathogenesis. In this paper, the molecular signaling pathways in the NPC are discussed for the holistic view of NPC development and progression. The important insights toward NPC pathogenesis may offer strategies for identification of novel biomarkers for diagnosis and prognosis.

## 1. Introduction

Nasopharyngeal carcinoma (NPC) is a squamous epithelial cancer arising from the lateral wall surface of nasopharynx [1]. Unlike other head and neck cancer, NPC shows a clear regional and racial prevalence. The incidence of NPC is high in the southern region of China, the Southeast Asia, Alaska, and native Greenlanders [2–4]. The differences in geographic and ethnic distribution reflect the multifactorial etiology of NPC, including the Epstein-Barr virus (EBV) infection, ethnics, genetic susceptibility, environmental factors, and food consumption [5, 6].

According to the world health organization, NPC can be classified into 3 subtypes of microscopic histological patterns. These include (i) type I, keratinizing squamous cell carcinoma that shows predominant features of producing keratin proteins, (ii) type II, differentiated nonkeratinizing carcinoma, and (iii) type III, nonkeratinizing carcinoma with less differentiation. While the NPC type I is uncommon in endemic areas, types II and III are more common and have been shown to be closely related to EBV infection [7]. There are lines of evidence showing that EBV plays a critical role in transforming nasopharyngeal epithelial cells into invasive cancer cells.

At present, treatment of NPC is usually via radiotherapy. NPC is more sensitive to ionizing radiation than other cancers. However, the treatment success mostly depends on the tumor, node, and metastasis (TNM) stages classification [8], which tend to be in the advanced stages at the point of diagnosis because the primary anatomical site of cancer growth is located in the silent painless area. The 5-year survival rate of stages I and II NPC ranges from 72 to 90%. However, the 5-year survival rate of stages III and IV NPC are ~55% and 30%, respectively, mostly due to a relatively high incidence of locoregional recurrence or metastasis [9]. Moreover, NPC has a poor prognosis because of late presentation of lesions, poor understanding of the molecular mechanisms, no suitable markers for early detection, and poor response to available therapies [10].

**Figure 1:**
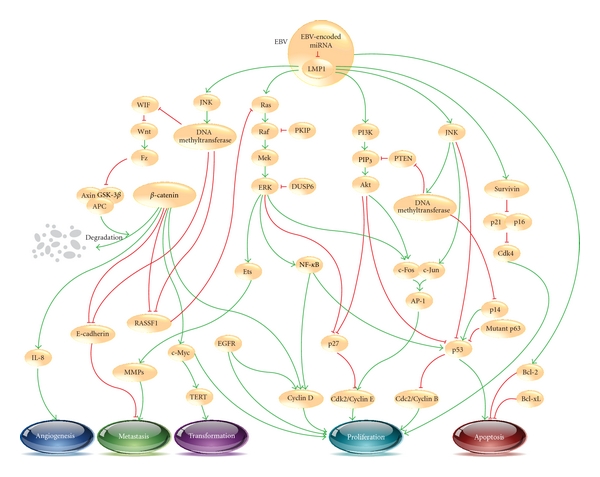
Overview of the signaling pathways in the pathogenesis of nasopharyngeal carcinoma (NPC). Initiation of upstream signaling proteins in the NPC development begins with LMP1. Subsequent induced activity of downstream proteins in several pathways such as *β*-catenin, NF-*κ*B, and AP-1 leads to dysregulation of cell proliferation (cdk/cyclin protein), cell transformation (TERT), increase in angiogenesis (IL-8) and metastasis (E-cadherin, MMPs), and inhibition of apoptosis (Bcl-2, p53). Green arrows and red blunt-end arrows represent up- and downregulation, respectively.

One elemental factor mediating the biological behaviors of NPC including carcinogenesis is the alteration of intracellular compartment signaling. Such signaling pathways are critical for cell survival, growth, and metastasis. However, research on the molecular signaling pathways in NPC development is in its infancy, when compared to other cancers such as breast cancer, colorectal cancer, and squamous cell carcinoma of neck and neck cancer. This paper explores the subject of the molecular signaling pathways in the NPC pathogenesis. The schematic representation of the pathways discussed in this paper is shown in Figure 1. The better understanding of the molecular signaling pathways in NPC may provide a substantial opportunity for identification of novel diagnostic and prognostic biomarkers and might also improve individual treatment in patients with NPC.

## 2. Wnt Signaling Pathway

 The Wnt signaling pathway is a protein network participating in multiple developmental processes of embryo and tissue homeostasis of adults [11]. The canonical Wnt pathway occurs when Wnt proteins interact with cell-surface receptors in the frizzled (Fz) family [12, 13], thereby activating the dishevelled (DSH) family proteins [14]. DSH is a key component of a membrane-associated Wnt receptor complex, which inhibits a bundle of proteins that includes axin, glycogen synthase kinase-3*β* (GSK-3*β*), and the adenomatous polyposis coli (APC) protein. The axin/GSK-3*β*/APC complex normally phosphorylates *β*-catenin, leading to its ubiquitin-mediated proteolytic degradation [15, 16]. Following the inhibition of the axin/GSK-3*β*/APC complex by Wnt signaling, a pool of cytoplasmic *β*-catenin is stabilized and translocates into the nucleus, thereby interacting with various transcription factors to promote specific gene expression, causing cellular proliferation and differentiation [11]. Furthermore, the cytoplasmic *β*-catenin can also bind to the intracellular domain of E-cadherin to maintain cellular adhesion in the normal cells [17, 18].

 Dysregulation of the Wnt signaling pathway has been found in many types of cancer including lung cancer, colorectal cancer, leukemia, and head and neck cancer [19–22]. Prolonged Wnt signaling activates DSH to phosphorylate GSK-3*β* resulting in its inactivation, in turn leading to *β*-catenin accumulation [23]. There are a number of reports suggesting the relevance of the Wnt signaling in the NPC development. The upregulation of frizzled receptor family 7 (*FZD7*) and downregulation of axin-2 (*AXIN2*) have been found in the NPC transcriptomics studies [24]. Moreover, the expression of an endogenous Wnt inhibitory protein, Wnt inhibitory factor (WIF), has been found to be decreased in NPC [25–27]. The *WIF* expression has been shown to be blocked via hypermethylation of its promoter in NPC cell lines [28]. The *WIF* promoter methylation levels relate to TNM stages [29]. These results indicate that abnormal Wnt signaling is common event in the NPC development.

 Prolonged activation of *β*-catenin induces the accumulation of intranuclear *β*-catenin in NPC cells [30], hinting that nuclear *β*-catenin is one of significant components of NPC development. GSK-3*β* can be inactivated by EBV infection leading to an increase in the level of cytoplasmic *β*-catenin in lymphocytes [31]. The downregulation of GSK-3*β* in NPC cells may be resulted from the upstream signaling pathways regulation such as Wnt or Akt pathways as well [19, 32]. Moreover, the levels of phosphorylated GSK-3*β* and intranuclear *β*-catenin have been shown to be higher in NPC cells after EBV infection [30]. The expression of *β*-catenin has been associated with advanced stages of NPC and inversely relates with the survival rate of patients [33]. It implies the important role of Wnt signaling pathway on the dysregulation of *β*-catenin in NPC.

*β*-catenin is able to interact with transcription factors and promotes gene expression which involves in the cancer development. It can activate several downstream proliferative signaling molecules such as c-Myc and cyclin D1 in cancer [19, 34]. Cyclin D is accountable for cell cycle progression through G1 phase. Overexpression of cyclin D1 allows cells with damaged DNA or chromosome to proceed through S phase without DNA damage repair, enhancing the risk of cancer development [35, 36]. It has been shown that the NPC cells exhibit overexpression of cyclin D1, which can be comparable to the expression level in head and neck squamous cell carcinomas (HNSCCs). The level of cyclin D1 is related to the local disease recurrence and sensitivity to the radiotherapy of head and neck cancer including NPC [37, 38]. However, the cellular consequences of cyclin D1 upregulation in NPC have yet to be determined.

 Furthermore, *β*-catenin can also interact with other proteins that have been linked to NPC carcinogenesis including (i) the interleukin-8 (*IL-8*), the molecule which has been shown to be an angiogenic factor in NPC [39], (ii) the tumor suppressor RAS association family 1A (RASSF1A), in which downregulation causes abnormal mitotic spindles, aneuploidy, and transformation of NPC cells [40], and (iii) E-cadherin, forming a complex with cytoplasmic *β*-catenin to maintain cellular adhesion [17], mediating cell communication and suppressing metastasis. Lower levels of the cytoplasmic *β*-catenin in the NPC accelerate the NPC progression and metastasis [18, 26]. Both mRNA and protein levels of E-cadherin in metastatic NPC cells have been shown to be lower comparing to the primary NPC [41, 42] or noncancerous cells [43]. Although several reports indicate the relationship of the Wnt signaling pathway and *β*-catenin activity in NPC development, however the detailed interaction of individual factors in the Wnt pathway have not been completely understood.

## 3. PI3K-Akt Signaling Pathway

 The phosphoinositide 3-kinases (PI3K) are a group of enzymes involved in diverse cellular functions including cell growth, proliferation, differentiation, motility, survival, and intracellular trafficking [44, 45]. Many of these functions relate to the ability of class I PI3K to phosphorylate and activate a serine/threonine protein kinase B (Akt), in turn regulating cell proliferation and preventing apoptosis [46]. Uncontrolled regulation of PI3K is therefore involved directly in cancer. Hyperactivation of PI3K pathway through various mechanisms is significant to the development of NPC. One of such mechanisms might be upregulation of PI3K catalytic subunit (*PIK3CA*) as it has been evident in head and neck cancer [47]. The EBV-encoded latent membrane protein 1 (LMP1), a key effector of EBV-mediated nasopharyngeal cell transformation [48–51], also directly activates PI3K, leading to Akt phosphorylation and activation of several downstream signaling [52], which includes the degradation of a cyclin-dependent kinase inhibitor p27, resulting in progression of cell cycle [48]. *c-Fos*, which encodes an oncogenic protein that binds to c-Jun protein to form the transcription factor AP-1, an essential regulator for cell proliferation and survival [53], is also upregulated in NPC via Akt activity [54]. Alterations of the *PI3K* gene at the genomic level, such as mutations and gene amplification, have been found to be strongly related with the distant metastasis, lymph node involvement, advanced tumor stages, as well as worse prognosis [44]. In addition, treatment of NPC cells with a PI3K inhibitor LY294002 results in inhibition of Akt activation, thereby hindering cell proliferation and inducing cell apoptosis [55]. Another potential mechanism of PI3K activation might be through the reduction in the phosphatase and tensin homolog protein (PTEN) [56]. Downregulation of PTEN has been observed in more than half of NPC cases [57]. Interestingly, comparison between the cancer cells and normal neighboring cells demonstrates that NPC cells exhibit the high level of Akt with the low level of PTEN expression [56]. The level of PTEN downregulation in the NPC type I has been shown to be greater than type II and III with poorly differentiated cells [57]. The mechanism of PTEN downregulation in NPC is still unclear but it might be as a result of the epigenetic alterations to *PTEN* at transcription level. Hypermethylation of *PTEN* promoter has been demonstrated in certain cancers such as laryngeal and thyroid cancer [58]. Although a number of gene promoter hypermethylation in NPC have been identified, the *PTEN* promoter hypermethylation has not been investigated [59]. On the other hand, nicotine, one of the environmental factors for NPC carcinogenesis [5], is recently found to stimulate PTEN degradation by phosphorylation the C-terminal of PTEN protein in lung cancer [60]. PTEN level has also been related with cancer aggressiveness. Downregulation of PTEN is frequently found in the stages III-IV of NPC, but usually not in the stages I-II [57]. Altogether, these data suggest that the Akt activation and/or PTEN inhibition lead to dysregulation of multiple cellular functions and have been closely associated with the NPC development and metastasis.

## 4. MAPK Pathway

 The mitogen-activated protein kinase (MAPK) pathway is a chain of proteins in the cell which communicates a signal from a receptor on the surface of the cell to the nucleus by phosphorylation of various transcription factors [61, 62]. The signals are transmitted through a cascade of kinases [63]. The MAPKs such as c-Jun N-terminal kinase (JNK) and extracellular signal-related kinase (ERK) have been shown to play an important role in cancer development [61].

 JNKs are also known as stress-activated protein kinases that are involved in cell survival and cell death. Normally, prolonged activation of JNK results in cellular apoptosis whereas transient activation leading to cellular survival and proliferation [64, 65]. Downregulation of *JNK* has been evident in cancer cells with tolerance for cell death [66]. Interestingly, unlike most cancers, NPC exhibits induced regulation of JNK via LMP1-dependent route [67–70]. Prolonged JNK activation has been evident in NPC as well as oral squamous cell carcinoma [71, 72]. Constitutive activation of JNK in NPC has a significant effect in cancer development including p53 inactivation via phosphorylation, activation of DNA methyltransferase leading to reduction in E-cadherin gene expression [68, 71]. However, the JNK overactivation pattern and its role in NPC is still unclear.

 ERKs are constitutively expressed MAP kinases that function in a variety of cellular regulation leading to cell growth and development [73]. Phosphorylation of ERK proteins via the Ras/Mek/ERK pathway cascade induces the activation of transcription factors NF-*κ*B, AP-1, and ETS [74], resulting in the downstream outputs including the induction of c-Fos, cyclin D1, and c-Myc, which are important in cellular proliferation and growth regulation [75, 76]. Upregulation of ERK has been found in NPC [77] as well as several types of cancer such as gastric adenocarcinoma [78], hepatocarcinoma [79], and renal cell carcinoma [80]. The mechanism of aberrant ERK activation has been reported to be involved with abnormality of upstream proteins in most types of cancer [81]. Up to 90% of pancreatic cancer exhibits the RAS mutations and the BRAF mutations have been found in 66% of melanoma. Moreover, more than half of most carcinomas showed overexpression of the epidermal growth factor receptors (EGFRs), resulting in aberrant activation of the ERK signaling pathway [82]. In case of NPC, the upregulation of ERK can be mediated through several mechanisms. For example, downregulation of the Raf kinase inhibitory protein (RKIP), a protein that inhibits the Raf protein activity and its downstream cascade including ERK, has been observed in NPC cells and has also been reported as a metastasis suppressor. RKIP has also been associated with advanced clinical stages, poor prognosis, and radio-resistant phenotypes in NPC cells [83], pointing to the potential use of RKIP as a biomarker for NPC prognosis. However, further investigations must be warranted in order to verify this claim. Another possible mechanism that might activate ERK is through loss of dual-specificity phosphatase 6 (DUSP6), which has also been found in NPC cells and shown to induce tumor formation and metastasis *in vivo *[84]. Moreover, LMP1-dependent mechanism has been proposed to trigger ERK activation in NPC cells by direct stimulation of RAS protein [23, 77]. Constitutively active ERK protein has also been reported to phosphorylate and inactivate p27, a cell cycle regulator protein, allowing the CDK2/cyclin E complex to remain activated, hence enhancing cell entry to the S phase [85]. On the other hand, LMP1 silences the expression of RAS association domain-containing proteins (RASSF) via hypermethylation leading to prolonged RAS activation [40, 86]. Additionally, the high ERK expression has been correlated with shorter overall survival rates and severe disease development in NPC patients [87]. Collectively, these data indicate that ERK pathway is involved in NPC progression and individual players within pose as potential molecular markers for NPC prognosis.

## 5. Apoptosis Pathway

 Dysregulation of apoptotic signals is significantly involved in development of various types of cancer including NPC. The well-known case is the aberrant activation of an apoptotic-regulated protein, B-cell lymphoma 2 (BCL-2). BCL-2 is a human proto-oncoprotein located in the membranes of the nuclear envelope, endoplasmic reticulum, and in the outer membrane of mitochondria. Overexpressed BCL-2 protein in NPC has been reported in a higher percentage than other head and neck cancers [88]. The upregulation of *Bcl-2* mRNA has been found in several studies in NPC biopsies [29, 89, 90], and might be linked to the EBV-dependent mechanism [91]. BCL-2 expression in the EBV-positive NPC cells has been shown to be higher than the EBV-negative counterparts [88, 92]. It is noteworthy that the expression of *Bcl-2* in NPC can also be upregulated through the LMP1-independent mechanism due to the fact that silencing of LMP1 does not affect *Bcl-2* expression [48]. Although *Bcl-2* expression is not directly related to EBV infection, BCL-2 can function synergistically with LMP1 to advance more rapid cell growth than BCL-2 alone in NPC [93]. Upregulation of BCL-2 has been closely related to aggressive traits in NPC including lymph node involvement, metastasis, recurrence, and poor survival rates in NPC patients [29, 89, 90, 92]. Furthermore, patients with the NPC stages III and IV, which exhibited low levels of *Bcl-2* expression, were shown to have higher disease-free 5-year survival rate than those with high *Bcl-2* expression [94, 95]. These data imply the critical role of BCL-2 in the NPC pathogenesis; however, the exact molecular mechanism of Bcl-2/BCL-2 in NPC is still unclear.

Tumor suppressor protein p53, which responses to DNA damage, triggers activation of DNA repair proteins and induces cell cycle arrest. It is evident that p53 is suppressed in several types of cancer. Interestingly, p53 expression level significantly increases in NPC and relates to the size of the tumors [70]. Upregulation of p53 has been associated with the EBV infection and high levels of LMP1 [91, 96, 97]. LMP1 activates nuclear factor kappa-light-chain-enhancer of activated B cells (NF-*κ*B) via the binding of tumor necrosis factor receptor-associated factors (TRAFs) [52]. NPC exhibits the overexpression of NF-*κ*B [25, 98], resulting in activation of components in the proliferative and survival pathways including p53 protein [99]. The decrease of kinase activity of cell division cycle 2/cyclin B (CDC2/cyclin B) complex, which can also be regulated through the p53, and inducing cell cycle arrest at G2/M phase has been found in NPC cells [99]. Despite the high level of p53, it is not successful to encourage NPC cells to undergo apoptosis [100]. The reasons that might underline this phenomena include the presence of mutated form of p63 and/or downregulation of p14 protein [101]. p63, a homolog of p53, has a conserved DNA binding domain similar to p53 protein and also induces cellular apoptosis [102]. The mutated form of p63 binds the DNA target, thereby blocking the p53 and fails to induce cellular apoptosis due to the lack of N-terminal transactivating domain [103]. Loss of p14 protein expression due to promoter hypermethylation [59] results in p53 degradation via ubiquitin-mediated proteolysis, hence enhancing cell survival [101]. The discrepancy between the high level of p53 and the loss of p14 leading to p53 degradation may arise from the multifactorial etiology of NPC. The puzzle of p53 overexpression in NPC has yet to be investigated. Upregulation of p53 in NPC cells may be advantageous to NPC development due to resistant to cellular apoptosis by decreasing the activity of JNK pathway [70, 100, 104]. However, we cannot exclude other possibilities that this could be limited to certain NPC cases.

 Survivin is a member of the apoptotic inhibitors. The antiapoptotic activity of this protein is mediated by the microtubules in mitotic spindles and inhibition of caspase activation. Upregulation of survivin, both mRNA and protein, has been found in NPC, with higher percentages especially in stages III and IV NPC [91, 105]. Survivin expression and nuclear translocation are induced by EBV infection via LMP1-mediated mechanism [91, 106]. Binding of intranuclear survivin to cyclin-dependent kinase 4 (CDK4) releases the inhibitory complex of p21 and p16 leading to CDK4-dependent entry to the S phase protein transcription and S phase progression in NPC [106, 107]. Overexpression of survivin is related to poor prognosis whereas inhibition of survivin reduces NPC cell viability and enhances sensitivity of NPC to radiotherapy [25, 108, 109]. These data suggest that survivin is a critical regulator in NPC development and has potential prognostic implication in NPC.

 Telomerase is a reverse transcriptase that adds specific DNA sequence at 3′ end of telomere regions at the ends of eukaryotic chromosomes. Since up to 100–200 nucleotides at chromosome end are lost in every DNA replication cycle, telomerase is an important enzyme that utilizes their own RNA molecules as a template to elongate telomeres for telomere length maintenance and prevent constant loss of important DNA. Human telomerase reverse transcriptase (TERT) is significant to transform normal nasopharyngeal cells into NPC as its high activity has been reported in most NPC as well as other head and neck cancers [110]. Interestingly, constitutive expression of telomerase can induce the alteration of the primary nasopharyngeal epithelial cells into immortalized nasopharyngeal epithelial cell lines [111], pointing towards the importance of TERT in NPC transformation. Upregulation of TERT is induced by EBV infection via LMP1-mediated mechanism. LMP1 has been demonstrated to increase the c-myc expression and NF-*κ*B. Expression of TERT is regulated by the c-myc activity and NF-*κ*B mediates the translocation of TERT protein into nucleus [112, 113].

## 6. EGFR Pathway

 The epidermal growth factor receptors (EGFRs) are the members of tyrosine kinase receptors. Similar to other head and neck cancers, overexpression of EGFR in NPC is quite frequent and has been reported to be as high as 80% in primary NPC biopsies [114–117]. The high levels of EGFR expression have been detected in NPC patients with advanced stages [115, 118]. Overexpression of EGFR results in high activity of its downstream signaling cascades such as RAS/ERK signaling, giving rise to irregular cell proliferation [119]. EBV infection has been shown to stimulate the endocytosis of EGFR and translocation into the nucleus [52]. While cytoplasmic EGFR binds to cyclins D1 and E to induce the G1/S phase progression [120], intranuclear EGFR acts as a transcription factor to promote the expression of cellular proliferation components [52]. In contrast, the inhibition of EGFR signaling does not totally inhibit NPC proliferation [121], suggesting that other pathways might be also involved in NPC development.

## 7. miRNA in NPC

 Recently, a novel function of noncoding genes, microRNA (miRNA), has emerged to play a role in regulation of several cellular processes [122, 123]. miRNAs are 20–24 nucleotide-in length RNA molecules, which function in the posttranscriptional regulation that represses protein translation and/or induces RNA degradation via binding to complementary sequences on the target mRNAs, resulting in targeted gene silencing. Primary miRNAs are usually transcribed from introns or noncoding regions and are cleaved in the nucleus by Drosha enzyme to yield hairpin precursor miRNAs (pre-miRNAs). Pre-miRNAs are then translocated into the cytoplasm and are subsequently cleaved by RNase III Dicer, giving rise to miRNA. These miRNA fragments execute their regulatory role as element of the RNA-induced silencing complex (RISC) [122, 124].

 Up to 40 miRNAs have been reported to be expressed in the different parts of EBV genome [125, 126]. The main target of EBV miRNA is its oncogene LMP1 [127]. The overexpression of LMP1 protein may results in the inhibition of cell proliferation and increase in apoptosis [128]. Therefore, to prevent excessive LMP1 expression, inhibition of LMP1 on NPC by miRNA results in NPC cells resistant to the apoptosis. Regulation of LMP1 expression via miRNA can be used to explain for the observed inconsistency between LMP1 trasnscripts and protein expression [127]. Understanding the function of miRNAs may provide the biomarkers of NPC development for screening high-risk populations.

 In addition to EBV-encoded miRNA, some cellular miRNAs in NPC have also been reported to be differentially expressed, leading to alterations in cellular gene expression that affect various signaling pathways in cell proliferation and apoptosis. A study of large-scale miRNA profiling in NPC comparing to normal adjacent nasopharyngeal cells revealed 35 miRNAs whose expression levels were notably changed in NPC samples. For example, upregulation of oncogenic miR17-92 and miR-155 and downregulation of tumor suppressive miR-34 family, miR-143, and miR-145 have been demonstrated. Twenty-two significantly down-regulated miRNAs are predicted for collectively targeted in NPC pathogenesis and progression [129], including the Wnt signaling pathway, cell cycle progression, and apoptotic and survival pathways [129]. Consistently, downregulation of miR-29c has been reported in primary NPC cells compared to normal nasopharyngeal mucosa. Most of miR-29c targeted genes encode extracellular matrix proteins including laminin-*γ*1. These proteins have been reported to be involved in cancer cell metastasis [130]. The miRNA data suggest that both viral and host cell miRNAs have significant functions on the NPC development and progression.

## 8. Summary

 In the pregenomic eras, highly integrated and complex circuitry of molecular signaling in NPC pathogenesis was only partially understood. Over the past decade, the knowledge of the molecular mechanisms in NPC carcinogenesis has been rapidly accumulated. Dysregulation and abnormal protein expression of molecules in certain signaling pathways involved in cellular functions including proliferation, adhesion, survival, and apoptosis has been demonstrated in the NPC cells. Detailed information on the complex network in signaling pathway leading to a coordinated pattern of gene expression and regulation in NPC will undoubtedly provide important clues to develop novel prognostic and therapeutic strategies for this cancer. Refining molecular markers into clinically relevant assays may assist in the detection of NPC in asymptomatic patients, as well as stage classification and monitoring disease progression and treatments. Furthermore, selective regulation of particular proteins targeting cancer cell proliferation, invasion, and apoptosis is a hopeful prospect for future anticancer therapy that slow disease progression and improve survival.
